# Tennessee State Medical Society

**Published:** 1889-06

**Authors:** 


### TENNESSEE STATE MEDICAL SOCIETY.

The 56th annual meeting of the Tennessee State Medical Society was held at Nashville, April 30th, May 1st and 2d.

The following is a *résumé* of the proceedings:

The Society was called to order by the president, T. J. Happel. After some preliminary work the committee on legislation reported that a medical bill had been passed by the last legislature. After considerable discussion a committee was appointed to select three names to be presented to the Governor for appointment on the Board of Examiners. The committee selected the names of C. Deaderick, J. B. Murfree and D. D. Saunders.

The secretary reported 254 members in good standing previous to the meeting.

The address of welcome was deliverd by Rev. Dr. G. W. F Price, at the Vendome Theatre, followed by the address of the president on "Alcohol in Medicine." This was followed by a concert furnished by local talent. Dr. Duncan Eve, of Nashville, was elected president, and D. E. Nelson, of Chattanooga, secretary.

Memphis was selected as the next place of meeting on the second Tuesday in April, 1890.

The following papers were presented:

"Typho-Malarial Fever," F. M. Duke, Wartrace.

"Importance of the Microscope in the Practice of Medicine and Surgery," James E. Reeves, Chattanooga.

"Physiological Action of Alcohol," R. F. Keys, Nashville.

"Recent Additions to our Pharmacopœia," T. A. Atkinson, Nashville.

"Tubercular Meningitis," J. R. Rathwell, Chattanooga.

"Diseases Peculiar to Gestation," J. B. Murfree, Murfreesboro.

"Asphyxia Neonatorum," C. W. Beaumont, Clarksville.

"Report of Four Abdominal Sections," W. D. Haggard, Nashville.

"Hysterectomy," Richard Douglas, Nashville.

"Some Uterine Displacements not Curable. What shall we do with them?" W. F. Rochelle, Jackson.

"Bilious Pneumonia," J. A. Crook, Jackson.

"Laparotomy in Visceral, Gunshot and Incised Wounds," C. S. Briggs, Nashville.

"Extirpation of the Tonsils," C. H. Lovelace, Dulsedom.

"Report of Surgical Cases," W. B. Wells, Chattanooga.

"Report of Hip Joint Amputation," P. F. Eve, Nashville.

"Some Points in the Treatment of Gonorrhœa, with a description of an appropriate syringe," W. F. Rochelle, Jackson.

"The Wire Corset in the Treatment of Spinal Affections, with Exhibit," A. J. Swaney, Gallatin.

"Report of a case of Self-castration and a case of Ovariotomy, with Complications," C. N. Cooper, Cleveland.

"Heterophonia," G. S. Savage, Nashville.

"The most common Eye Diseases and their Treatment," J. G. Sinclair.

"New Instruments—Tongue depressor, Improvised lid elevator, method of clearing Secretions from Trachea and larger Bronchi after Tracheotomy," Frank Trester Smith, Chattanooga.

The attendance was the largest of any meeting in the history of the Society, 156 members having registered.

The meeting was a success scientifically, financially and socially.

